# Friendships in Emerging Adulthood: The Role of Parental and Friendship Attachment Representations and Intimacy

**DOI:** 10.1177/01461672231195339

**Published:** 2023-09-05

**Authors:** Marie G. Oldeman, Antonius H. N. Cillessen, Yvonne H. M. van den Berg

**Affiliations:** 1 Radboud University, Nijmegen, The Netherlands; 2 University of Amsterdam, The Netherlands

**Keywords:** emerging adulthood, attachment, actor–partner interdependence model, friendship, intimacy

## Abstract

The current studies addressed the associations between attachment representations with parents and a single best friend, intimacy behaviors (self-disclosure and support-seeking), and friendship quality in emerging adulthood, using the actor–partner interdependence mediation model (APIMeM). Study 1 (*N* = 186 dyads) examined whether attachment to parents predicted friendship quality, and whether this was mediated by attachment to their best friend. More avoidance or anxiety with parents predicted lower friendship quality, which was mediated by avoidance or anxiety with their best friend. Study 2 (*N* = 118 dyads) examined whether self-disclosure and support-seeking mediated the link between attachment with best friend and friendship quality. Anxiety with their best friend predicted lower friendship quality, which was mediated by support-seeking. Anxiety predicted less self-disclosure and support-seeking. We found no effects of avoidance. No partner effects were found in both studies. The findings are discussed in terms of adult attachment theory.

Friendships are essential relationships in most people’s lives: they serve as cognitive and emotional resources, they aid in reaching developmental tasks, and they are related to greater well-being and even physical health (see, for reviews, Hartup & Stevens, 1997; Hojjat & Moyer, 2017). Especially in emerging adulthood, when important life events occur, such as moving out or getting involved in serious romantic relationships, friendships are important sources of intimacy and support (Fehr, 2004; Hojjat & Moyer, 2017). However, we know little about why some emerging adults succeed in forming high-quality friendships, whereas others form lower-quality friendships (Hojjat & Moyer, 2017).

Bowlby’s (1973, 1979) attachment theory has been influential in explaining individual differences in relationships across the lifespan (see also Cassidy & Shaver, 2016). Attachment theory is based on the idea that individuals form internal working models in their relationships, which influence the way they view themselves and others. The original theory and subsequent research emphasize the importance of working models formed with parents early in life, which influence attachment and relationships throughout the lifespan (Bowlby, 1973, 1979; Fraley et al., 2013; Steele et al., 2014).

During emerging adulthood, the attachments with parents and friends may change, as attachment-related functions are transferred from parents to friends (Fraley & Davis, 1997; Markiewicz et al., 2006). Therefore, the attachments in these relationships may be interdependent, that is, changes in attachment with parents may affect attachment with friends (Hojjat & Moyer, 2017). These changes in attachment may also affect friendship quality, as these changes are associated with intimacy and support in friendships (Fraley & Davis, 1997). However, research on the interplay between these current attachments with parents and friends, friendship behaviors, and friendship quality is scarce. Moreover, most attachment research has focused on romantic relationships in emerging adulthood or friendships in adolescence (e.g., Bartholomew & Horowitz, 1991; Markiewicz et al., 2001; Simpson et al., 1992; Zimmermann, 2004), despite the importance of friendships in emerging adulthood. The goal of the present studies therefore was to examine the interrelations between attachment with parents and friends, friendship behaviors, and friendship quality in emerging adulthood.

## Attachment in Emerging Adulthood

According to Bowlby (1973), the attachments we form in our relationships are crucial to the nature and quality of these relationships. Bowlby (1973, 1979) proposed that through repeated experiences with caregivers (usually, parents), children develop an internal working model of the self (i.e., the degree to which they judge themselves as worthy of love and support) and an internal working model of others (i.e., the degree to which they judge other people as available and trustworthy) (see also Collins & Read, 1990). The theory states that early experiences with parents affect future relationships through these working models, as they affect individuals’ cognitions, emotions, and behaviors in current and future relationships (Bowlby, 1973; Collins & Read, 1990).

This does not mean that early working models are destiny. Importantly, working models can be revised over the life course as a result of new social experiences. This was supported by longitudinal studies that demonstrated that early attachment experiences with parents are only moderately correlated with attachment with parents, friends, and romantic partners in adulthood (see, for example, Fraley et al., 2013; Steele et al., 2014; Zayas et al., 2011). Moreover, it has been proposed that current relationships reflect current working models and not early working models, although early working models may have shaped current working models (Berlin & Dodge, 2004). It is therefore valuable to not only examine associations between early parental attachment and friendships, but also examine associations between current attachment representations with parents and friends to understand friendships in emerging adulthood.

Researchers have proposed that attachment in (emerging) adulthood consists of two dimensions: anxiety and avoidance (Brennan et al., 1998; Mikulincer et al., 2003; Simpson et al., 1992). Attachment-related anxiety relates to the working model of the self and reflects a fear of rejection, an excessive need for approval, and distress when the other person is unresponsive or unavailable. Attachment-related avoidance relates to the working model of others and reflects a strong need for independence and a fear for or aversion of intimacy (Bartholomew & Horowitz, 1991; Collins & Read, 1990; Wei et al., 2007).

Furthermore, theory proposes that adult attachment is hierarchically organized. That is, adults are seen as having a global attachment representation (i.e., general attachment representations across different types of relationships), domain-specific attachment representations (i.e., attachment representations within specific relationship types, such as friendships), and relationship-specific attachment representations (i.e., attachment representations with a specific person) (Collins & Read, 1994; Pierce & Lydon, 2001; Sibley & Overall, 2008). All these attachment representations together are expected to predict the quality and intimacy of relationships with others (Pierce & Lydon, 2001). As a result of this hierarchical organization, adults can have different attachment representations across relationship domains (e.g., different attachment representations for friendships and parental relationships) and even across a specific relationship domain (e.g., different representations for two individual friends) (Bohn et al., 2023; Overall et al., 2003; Sibley & Overall, 2008).

## Attachment and Friendships

As a result of the hierarchical structure of attachment representations, attachments to parents and friends may or may not overlap in emerging adulthood. Moreover, since emerging adults transfer attachment-related functions (seeking proximity, using others as a safe haven or secure base) from parents to peers (Fraley & Davis, 1997; Markiewicz et al., 2006), concurrent attachment representations with parents may play an important role in shaping attachment representations with friends, and subsequently, the quality of friendships. However, it is unclear whether these dependencies lead to similar or different attachment representations in emerging adults’ relationships with parents and friends.

On one hand, emerging adults may form similar attachment representations with parents and a friend. The hierarchical structure of attachment in adulthood may imply overlap due to the same underlying global attachment representation. For example, if an individual is avoidant and generally believes that other people are untrustworthy, this individual may show avoidant attachment representations with parents and a friend. In addition, overlap might exist because attachment representations with parents may be directly transferred to the relationship-specific attachment representations with a best friend when emerging adults transfer attachment-related functions from parents to peers (Fraley & Davis, 1997; Markiewicz et al., 2006). This would result in similar attachment representations with parents and a friend. This consistency has been reported by Bohn et al. (2023), Fraley and Dugan (2021) and Furman et al. (2002), and they also acknowledge differences between attachment representations to parents and relationship-specific attachment representations to a best friend.

On the other hand, emerging adults may thus form different attachment representations with parents and their friend. The hierarchical structure also allows for less overlap between different relationship-specific attachment representations. Relationship-specific attachment representations depend not only on one’s global attachment representation, but also on characteristics and cognitions about a specific relationship type and the partner (Bohn et al., 2023; Overall et al., 2003). Consequently, an individual can have insecure attachment representations with parents, but secure attachment representations with their best friend. According to a compensation model, individuals might compensate for insecure attachment representations and low-quality relationships with parents by forming strong attachments and high-quality relationships with friends (Helsen et al., 2000; Nada Raja et al., 1992). As a result, insecure attachment representations with parents would not be transferred to friendship attachment representations, and hence, emerging adults may still form supportive, high-quality friendships.

It is important to examine these two opposing hypotheses, as the attachment representations with both parents and a friend may play a crucial role in the quality of emerging adults’ friendships. In adolescence, attachment with parents has been linked to friendship quality (Furman, 2001; Zimmermann, 2004). In emerging adulthood, secure attachment with friends has been linked to more supportive and higher-quality friendships (Asendorpf & Wilpers, 2000; Özen et al., 2010). However, no studies have examined emerging adults’ attachment representations with both parents and their best friend in relation to their friendship quality. Yet, it is crucial to examine how these representations interact and relate to friendship quality, as the continuity and compensation perspective pose different hypotheses that might explain why some emerging adults develop high-quality friendships, whereas others develop low-quality friendships.

## Study 1

The goal of our first study was to examine how concurrent attachment representations with parents are related to attachment representations with a best friend and friendship quality. Because both attachment and friendships are dyadic processes, it is important to take an interdependence perspective (Kelley & Thibaut, 1978; Kenny et al., 2006). That is, emerging adults, their parents, and their best friend mutually influence each other and thereby directly affect each other’s relationship processes and outcomes, including attachment representations and perceived friendship quality (Kenny et al., 2006). Previous research in this domain has often neglected the dyadic perspective, but it is crucial to fully understand relationships. Therefore, we used the actor–partner interdependence mediation model (APIMeM; Ledermann et al., 2011), which assumes that relationship outcomes depend on an actor’s characteristics (actor effects) as well as a partner’s characteristics (partner effects) (Kashy & Kenny, 2000; Kenny et al., 2006). It might be that individuals who have insecure attachment representations with their parents may have different relationship expectations and may behave differently, and as a result their friend might form a more insecure representation to them and experience low friendship quality. For example, individuals who have more insecure attachment representations to their parents seek less emotional support from their friends (Zimmermann, 2004). As a result, they might seek out a best friend who has similar attachment representations and therefore relationship expectations and behaviors, or their best friend might form a relationship-specific insecure attachment representations to their best friend as a result of their friend’s behavior (e.g., forming avoidant attachment representations because their friend is unresponsive).

Based on current theories and findings, we formulated competing hypotheses. First, in line with the continuity perspective (e.g., Furman et al., 2002), we hypothesized that more insecure parental representations (i.e., more avoidance or anxiety) are associated with more insecure friendship representations and with lower friendship quality as experienced by emerging adults themselves (i.e., actor effect) as well as by their best friend (i.e., partner effect). Second, in line with the compensation perspective (Helsen et al., 2000; Nada Raja et al., 1992), we hypothesized that insecure parental representations can be compensated for by forming secure representations with their best friend, and a subsequently higher-quality friendship as experienced by emerging adults themselves (i.e., actor effect) and their best friend (i.e., partner effect). We hypothesized no actor nor partner effects from anxiety to avoidance, as anxiety and avoidance are two separate regions of the attachment continuum with different and sometimes opposite biological, cognitive, affective, and behavioral processes (Simpson & Rholes, 2017).

## Method

### Participants and Procedure

The preregistration for this study, including the study design, power analysis, inclusion criteria, planned analyses, and codebook (including the description of all other measured variables) can be found at https://osf.io/uk365/. Because we were not aware of the possibility to use Monte Carlo simulations to determine power for an APIMeM at the time we conducted our initial power analyses, we performed a sensitivity power analysis for an actor–partner interdependence model without mediation using APIMpower (Kenny, 2018). Our sample afforded power to detect moderate effect sizes when treating dyads as indistinguishable and assuming moderate correlations between the study variables, Cohen’s *d* = .30, α = .05 (two-tailed), 1–β = .82. Our post hoc power analysis using Monte Carlo simulations (Ledermann et al., 2022) showed adequate power (1–β ≥ .80) for most direct actor and indirect actor–actor effects, but low power for the partner effects (1–β < .80).

Participants were part of the That’s What Friends Are For project, a longitudinal online questionnaire study of relationships and socio-emotional well-being among emerging adults (van den Berg, 2021). This project consisted of three cohorts (Cohort 1 in 2018 through Cohort 3 in 2020). Participant dyads were recruited via the university’s research participation system and the personal network of the student assistants. Participants were rewarded with a €5 voucher or 1 study credit. The study procedure was approved by the Institutional Review Board of the Faculty of Social Sciences at Radboud University (ECSW-2018-025R1).

In total, 560 participants (280 dyads) expressed interest in participation and received the link to the online questionnaire. Of those, 108 participants did not respond to our online invitation and reminders or withdrew participation. From the remaining dyads (452 individual participants), we selected the data from dyads in which both friends completed at least one questionnaire relevant for the current research questions. This resulted in a final sample of 186 dyads (372 participants, *M*_age_ = 21.86, *SD*_age_ = 2.33, range = 18-33, 80% female). This final sample did not differ from the total sample on the demographic variables (*M*_age_ = 21.77, *SD*_age_ = 2.27, 78% female). Most participants were university students (67%), followed by applied university students (15%), emerging adults currently not in school (13%), and others (5%). Most dyads were same-gender (83%; 132 female–female and 22 male–male); 32 dyads were mixed-gender (17%). Friendship duration ranged from 5 months to 22 years.

### Measures

#### Attachment Representations

Participants completed the Dutch version of the Experiences in Close Relationships—Relationship Structures (ECR-RS) to measure attachment representations for mother, father, and best friend (Fraley, Heffernan, Vicary and Brumbaugh, 2011). The Dutch version consists of an extra item for the anxiety subscale compared with the original ECR-RS. The questionnaire uses a 7-point Likert-type scale (1 = *disagree strongly* to 7 = *agree strongly*) to assess the subscales avoidance (Items 1-6) and anxiety (Items 7-10). A sample item for avoidance is “I usually discuss my problems and concerns with my mother” (reverse coded). A sample item for anxiety is “I’m afraid that my mother may abandon me.”

Mean anxious and avoidant attachment representation scores were calculated for each attachment figure by averaging the items of each subscale, with higher scores indicating more anxiety or avoidance. The scores for mother and father were aggregated into overall scores for anxiety and avoidance score for parents, since we did not have different hypotheses for attachment with mother or father. The scales showed acceptable to good internal reliability for parents and best friend (*avoidance*: .78 ≤ α ≤ .89; *Anxiety*: .77 ≤ α ≤ .83).

#### Friendship Quality

Friendship quality was measured with the Network of Relationships Inventory—Relationships Quality Version (NRI-RQV), which has 30 items rated on a 5-point Likert-type scale (Buhrmester & Furman, 2008; Furman & Buhrmester, 1985). A sample item is “How happy are you with your relationship with your best friend?.” Participants reported on the same best friend as for the attachment measure. Items were averaged, with higher means indicating higher friendship quality (α = .88).

## Results

### Preliminary Analyses

All statistics were done in R (version 4.0.2; R Core Team, 2020). Preliminary analyses were performed using the “Hmisc” package (version 4.6.0; Harrell Jr, 2021) and the “pastecs” package (version 1.3.21; Grosjean & Ibanez, 2018). Table 1 presents the means and standard deviations as well as the correlations between the study variables. The statistical significance of the correlations was adjusted to account for the indistinguishability of the dyads, using the formulas by Griffin and Gonzalez (1995). The within-person correlations (i.e., correlations among study variables within dyad members) are shown above the diagonal; the between-person correlations (i.e., correlations among study variables between dyad members) are shown below the diagonal; intraclass correlations (i.e., correlations of each study variable between Friend 1 and Friend 2) are shown on the diagonal.

**Table 1.** Means, Standard Deviations, and Correlations of Study 1 Variables.

Variables	1.	2.	3.	4.	5.
1. Avoidance with parents	.22**	.55***	.22***	.30***	−.15**
2. Anxiety with parents	.14**	.19**	.13*	.45***	−.20***
3. Avoidance with best friend	.00	−.06	.22**	.28***	−.59***
4. Anxiety with best friend	.10	.08	.05	.11	−.38***
5. Friendship quality	−.02	−.03	−.19***	−.11	.28***
*M*	3.25	1.61	2.14	1.95	4.13
*SD*	1.25	.90	.83	1.11	.33

*Note*. This table shows the within-person (above diagonal), between-person (below diagonal), and intraclass (on diagonal) correlations.

* *p* < .05. **p < .01. ***p < .001.

### Model Specification

An APIMeM was run using the “lavaan” package in R (version 0.6.9; Rosseel, 2012) to examine the effect of attachment representations with parents on friendship quality, mediated by attachment representations with best friend. Because best friends are indistinguishable dyad members, equality constraints were imposed on the actor effects, partner effects, predictor means and variances, mediator and outcome intercepts and residual variances, and covariances between friends’ error terms (Ledermann et al., 2011).

Upon specification of the model, the distribution of variables was checked using the “pastecs” package (version 1.3.21; Grosjean & Ibanez, 2018) for numerical inspection and the “lattice” package (version 0.20.41; Sarkar, 2008) for visual inspection. Although numerical inspection of the distributions showed no severe violations of normality (skew < 2; kurtosis < 7), we used the Yuan–Bentler correction because visual inspection showed that attachment representations with parents and best friend were slightly positively skewed.

### Initial Model Check

Before fitting our hypothesized model, we first compared an independent (null) model with a saturated APIMeM to determine whether the APIMeM improved model fit. To apply these models to indistinguishable dyads, degrees of freedom and fit indices were adjusted (Olsen & Kenny, 2006). In the interchangeable null model (I-NULL), only the means and variances of the observed variables were set equal for both friends, whereas the actor effects, partner effects, and covariances between the variables were constrained to 0. The I-NULL model showed poor fit: χ^2^(55) = 487.951, *p* < .001. In the interchangeable saturated model (I-SAT), all actor and partner effects, means and variances for the predictors, intercepts and residual variances for the mediators and outcome variables, and covariances were set equal for both friends. The I-SAT model showed good fit, χ^2^(55) = 487.951, *p* < .001, and significantly better fit than the I-NULL model, χ^2^(25) = 441.309, *p* < .001.

### Final Model Results

**Figure 1.**
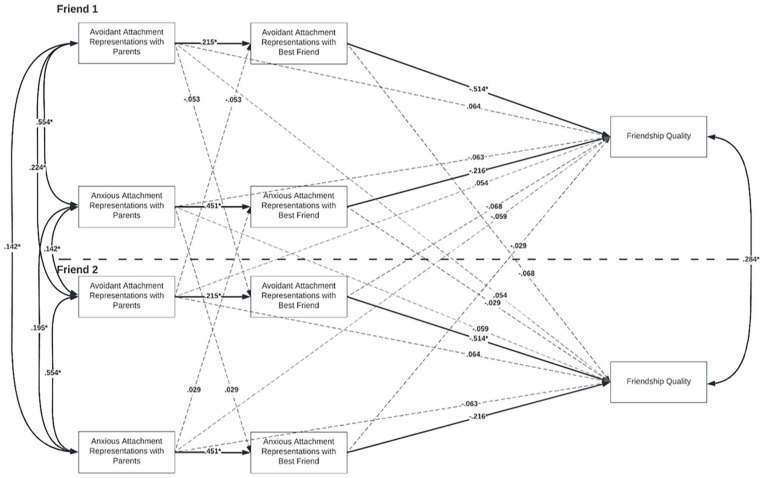
APIMeM of Study 1. *Note.* APIMeM of best friends’ attachment representations with parents and friendship quality, mediated by attachment representations with best friend. (Standardized) estimates are shown for all direct actor and partner effects, covariances between the predictors, and covariances between the outcome variables. The covariances between the mediators are not depicted in the model. *p < .05.

Because the initial model check was successful, we ran our hypothesized model. The degrees of freedom and fit indices were adjusted (Olsen & Kenny, 2006; see Figure 1). The hypothesized model showed good fit, χ^2^(34) = 4.66, *p* = .324, RMSEA = .030, CFI = .99. Table 2 presents all (un)standardized *direct* actor and partner effects and their corresponding 95% bootstrapped confidence intervals. Table 3 presents all (un)standardized *indirect* (mediated) actor and partner effects and their corresponding 95% bootstrapped confidence intervals.

**Table 2.** Direct Effects and Bootstrapped 95% Confidence Intervals From the APIMeM in Study 1.

Direct effect	Unst.	[95% CI]	Std.
*Actor avoidance*			
Actor avoidance with parents → actor avoidance with best friend	**.142**	**[.064, .220]**	**.215**
Actor avoidance with parents → actor friendship quality	.017	[−.011, .045]	.064
Actor avoidance with best friend → actor friendship quality	**−.204**	**[−.237, −.171]**	**−.514**
*Actor anxiety*			
Actor anxiety with parents → actor anxiety with best friend	**.560**	**[.386, .735]**	**.451**
Actor anxiety with parents → actor friendship quality direct effect	−.023	[−.062, .015]	−.063
Actor anxiety with best friend → friendship quality direct effect	**−.064**	**[−.097, −.030]**	**−.216**
*Partner avoidance*			
Actor avoidance with parents → partner avoidance with best friend	−.035	[−.100, .031]	−.053
Actor avoidance with parents → partner friendship quality direct effect	.014	[−.012, .040]	.054
Actor avoidance with best friend → partner friendship quality direct effect	−.027	[−.059, .005]	−.068
*Partner anxiety*			
Actor anxiety with parents → partner anxiety with best friend	.036	[−.084, .156]	.029
Actor anxiety with parents → partner friendship quality direct effect	−.022	[−.059, .016]	−.059
Actor anxiety with best friend → partner friendship quality direct effect	−.008	[−.038, .021]	−.029

*Note.* Unstandardized (Unst.) and standardized (Std.) estimates and bootstrapped confidence intervals are presented for all direct effects. Estimates in bold are statistically significant (*p* < .05).

**Table 3.** Indirect (Mediation) Effects and Bootstrapped 95% Confidence Intervals From the APIMeM in Study 1.

Effect	Unst.	[95% CI]	Std.
*Actor avoidance*			
Actor avoidance with parents → actor friendship quality total effect	−.011	[−.042, .018]	−.043
Actor avoidance with parents → actor friendship quality total indirect effect	**−.028**	**[−.044, −.012]**	**−.107**
Actor avoidance with parents → actor avoidance with best friend → actor friendship quality	**−.029**	**[−.045, −.013]**	**−.111**
Actor avoidance with parents → partner avoidance with best friend → actor friendship quality	.001	[−.001, .003]	.004
*Actor anxiety*			
Actor anxiety with parents → actor friendship quality total effect	**−.059**	**[−.093, −.025]**	**−.161**
Actor anxiety with parents → actor friendship quality total indirect effect	**−.036**	**[−.059, −.013]**	**−.098**
Actor anxiety with parents → actor anxiety with best friend → actor friendship quality	**−.036**	**[−.058, −.013]**	**−.096**
Actor anxiety with parents → partner anxiety with best friend → actor friendship quality	.000	[−.002, .001]	−.001
*Partner avoidance*			
Actor avoidance with parents → partner friendship quality total effect	.017	[−.011, .045]	.066
Actor avoidance with parents → partner friendship quality total indirect effect	.003	[−.011, .017]	.012
Actor avoidance with parents → actor avoidance with best friend → partner friendship quality	−.004	[−.009, .001]	−.015
Actor avoidance with parents → partner avoidance with best friend → partner friendship quality	.007	[−.006, .020]	.027
*Partner anxiety*			
Actor anxiety with parents → partner friendship quality total effect	−.029	[−.064, .007]	−.078
Actor anxiety with parents → partner friendship quality total indirect effect	−.007	[−.026, .012]	−.019
Actor anxiety with parents → actor anxiety with best friend → partner friendship quality	−.005	[−.021, .012]	−.013
Actor anxiety with parents → partner anxiety with best friend → partner friendship quality	−.002	[−.010, .006]	−.006

*Note.* Unstandardized (Unst.) and standardized (Std.) estimates and bootstrapped confidence intervals are presented for all indirect effects. Estimates in bold are statistically significant (*p* < .05).

#### Actor Direct Effects

Participants who reported higher anxiety with their parents reported higher anxiety with their best friend and participants who reported higher anxiety with their best friend reported lower friendship quality. Similarly, participants who reported higher avoidance with their parents reported higher avoidance with their best friend and participants who reported higher avoidance with their best friend reported lower friendship quality. The continuity of attachment representations was larger for anxiety than for avoidance, but avoidance with best friend had a larger effect on friendship quality than anxiety.

#### Partner Direct Effects

Contrary to our expectations, no significant partner effects emerged. Anxiety or avoidance with parents nor with best friend thus did not predict their friend’s perceived friendship quality.

#### Mediation Effects

The significance test of the mediation effects was based on the estimation of the indirect effect with corresponding standard errors and bias-corrected bootstrapped confidence intervals. Two small but significant actor–actor mediation effects emerged. First, the association between avoidance with parents and friendship quality was mediated by avoidance with best friend. Second, the association between anxiety with parents and friendship quality was mediated by anxiety with best friend. Thus, participants who reported higher levels of avoidance or anxiety with their parents also reported higher levels of avoidance or anxiety with their friend, and in turn experienced lower friendship quality.

### Supplemental Analyses

In our primary analyses, we aggregated the anxious attachment representations and avoidant attachment representations for mother and father into an anxious attachment representation variable and avoidant attachment representations variable for parents. We conducted two supplementary analyses to examine whether the pattern of results differed for mother and father separately. We found a very similar pattern when we used anxious and avoidant attachment representations to mother and father separately. The significance of the effects nor effect sizes did not differ when combining anxiety and avoidance to parents or when examining anxiety and avoidance to mother or father separately. The results are presented in Supplementary Tables 1 to 4.

## Summary

Consistent with the continuity perspective, more avoidant or anxious attachment representations with parents predicted more avoidant or anxious attachment representations with a best friend, which in turn predicted lower friendship quality. The results did not support the compensation perspective. We found no partner effects, indicating that attachment representations with parents or best friend did not predict how the other friend experienced the friendship quality. Thus, attachment representations with parents and best friend predicted one’s own perceived friendship quality. In the next study, we zoom in on the association between attachment representations with best friend and friendship quality and examine whether intimacy behaviors may mediate this association.

## Study 2

Previous literature has emphasized that intimacy behaviors, such as self-disclosure and support, differ between secure and insecure individuals and underly the link between attachment representations and relationship quality (Collins & Read, 1990; Markiewicz et al., 2001; Zimmermann, 2004). These behaviors become increasingly important during adolescence and are the hallmark of high-quality adult friendships (Hartup & Stevens, 1997).

As individuals who have low anxious and avoidant friendship attachment representations are comfortable with intimacy, they likely engage in self-disclosure and support-seeking (Bartholomew & Horowitz, 1991; Cassidy & Shaver, 2016; Fraley, Heffernan, Vicary & Brumbaugh, 2011). In contrast, individuals who are more avoidant or anxious with their friends, might not be comfortable with intimacy and consequently show atypical levels of disclosure and support, resulting in lower friendship quality (Bartholomew & Horowitz, 1991; Cassidy & Shaver, 2016; Fraley, Heffernan, Vicary & Brumbaugh, 2011). For avoidant individuals, it has been found that they seek less emotional support in romantic relationships (Simpson et al., 1992), rate themselves as more emotionally independent from others (Zimmermann, 2004), and do not value emotional support in friendships (Zimmermann, 2004).

For anxious individuals, the pattern of findings is more complex. On one hand, individuals high in anxiety might seek excessive emotional support and self-disclosure in friendships, as they have a strong desire for close and supportive relationships (Collins & Read, 1990; Mikulincer et al., 2003). On the other hand, they might avoid intimate behaviors (Mikulincer & Nachson, 1991), as they judge themselves to be unworthy of support and want to avoid possible rejection by their friend (Bartholomew & Horowitz, 1991). These competing predictions have both received empirical support. In some studies, anxious individuals sought less emotional support and showed less self-disclosure than secure individuals (Florian et al., 1995; Grabill & Kerns, 2000). Other studies showed higher levels of self-disclosure (Grabill & Kerns, 2000), or no differences (Mikulincer & Nachson, 1991; Simpson et al., 1992). Thus, research has shown that avoidant attachment representations are related to lower levels of intimacy, but it is unclear how anxious attachment representations affect intimacy behaviors.

In Study 2, we aimed to further examine these predictions and examine whether intimacy behaviors mediate the association between friendship attachment representations and friendship quality in a dyadic diary study. Based on previous literature (Bartholomew & Horowitz, 1991; Cassidy & Shaver, 2016; Florian et al., 1995; Fraley, Heffernan, Vicary & Brumbaugh, 2011; Grabill & Kerns, 2000; Simpson et al., 1992; Zimmermann, 2004) and the results of Study 1, we hypothesized that emerging adults with more avoidant friendship attachment representations would experience lower friendship quality as they disclose less and seek less emotional support from their friend (actor effects). Furthermore, we hypothesized that emerging adults with more anxious friendship attachment representations would experience lower friendship quality, either because they disclose less and seek less support from their friend, or because they excessively disclose and seek support from their friend (actor effects). Although we found no partner effects in Study 1, we hypothesized that emerging adults would experience lower friendship quality when their friends have more anxious or avoidant attachment representations, because their friend discloses less and seeks less support from them (anxious/avoidant) or because their friend discloses and seeks support excessively (anxious).

## Method

### Participants and Procedure

The preregistration for this study, including the study design, planned sample size, inclusion criteria, planned analyses, and codebook (including the description of all other measured variables) can be found at https://osf.io/uk365/. Our target sample size for Study 2 was 160 participants (80 dyads). Power-analyses based on APIMpower (Kenny, 2018) showed sufficient power to detect moderate effect sizes when treating dyads as indistinguishable and assuming moderate correlations between the study variables, Cohen’s *d* = .45, α = .05 (two-tailed), 1–β = .805. However, we decided to collect more data until we ran out of resources. Our post hoc power analysis using Monte Carlo simulations (Ledermann et al., 2022) showed adequate power (1–β ≥ .80) for most actor and some partner effects, but low power for some indirect actor and partner effects (1–β < .80).

Participants signed up via the university’s research participation system. They were invited to send the researcher contact details of the best friend with whom they wanted to participate and to indicate in which week they wanted to start the questionnaires. Each week, a new cohort started, but not during the Christmas break. This way, participants could schedule their participation when they would have the time and not participate during major events (e.g., Christmas, exam periods), as recommended by Reis and Wheeler (1991). Data collection took place between October 2021 and February 2022.

When participants had sent the contact details of their friend, they both received the first questionnaire. In this questionnaire, participants reported on several demographic variables, potential involvement with a romantic partner, their attachment representations, and friendship quality. Participants had 3 to 4 days to complete this questionnaire and were sent a reminder if they had not yet completed the questionnaire before the daily diary period started. During the daily diary period, participants received the daily questionnaire at a fixed time for 2 weeks, as this represents a generalizable and accurate reflection of social activity without being too taxing for participants (Reis & Wheeler, 1991). After the diary period, participants completed a final questionnaire about their friendship quality and their experiences with the daily diary. Upon completion of the questionnaires, participants received 1.5 course credit or a €15 voucher. Moreover, dyads were entered into a raffle to win two €25 vouchers if both dyad members had completed the questionnaires. The study procedure was approved by the Institutional Review Board of the Faculty of Social Sciences at Radboud University (ECSW-2021-113).

In total, 254 individuals (127 dyads) expressed interest in participation and received the links to the online questionnaires. Seven dyads were excluded because one or both members did not fall within the intended age range (18–29 years). Two additional dyads were excluded because one member did not complete any of the measures. Of the remaining 118 dyads (236 individuals), 108 dyads (92%) completed all relevant measures, seven dyads consisted of one member with complete data and one member who completed at least one relevant measure, and three dyads consisted of two members who completed at least one relevant measure.

The average age of the participants was 20.68 years (*SD*_age_ = 2.62, range = 17–28) and the majority was female (82%). Most participants were university students (67%), followed by applied university students (15%), emerging adults currently not in school (13%), and others (5%). Most dyads were same-gender (75%; 82 female–female and 7 male–male); 29 dyads were mixed-gender (25%). Friendship duration ranged from 2 months to 20 years.

### Measures

#### Attachment Representations

Attachment representations were measured as described in Study 1. For this study, only the attachment representations with best friend were used. The scales showed good internal reliability for best friend (*avoidance*: α = .80; *anxiety*: α = .83).

#### Rochester Interaction Record

The Rochester Interaction Record (RIR; Reis & Wheeler, 1991; Wheeler & Nezlek, 1977) was adapted for the purpose of this study. Participants were asked to record each day whether they interacted with their best friend, and if so, the duration and context of their interactions. Participants were also asked to report the amount of self-disclosure (by themselves and their friend) and emotional support-seeking and provision on a 5-point Likert-type scale (1 = *never*; 5 = *a great deal*). Participants were instructed to record both online and offline interactions. To ensure the reliability and validity of the interaction records, the questions included definitions of self-disclosure and emotional support based on those by Langford et al. (1997) and Ignatius and Kokkonen (2007). Self-disclosure was defined as “any personal or private information that you reveal about yourself to your best friends (including thoughts, feelings and experiences).” Emotional support was defined as “any way by which you or your best friend communicates care and concern, offers reassurance, empathy, comfort, and acceptance.”

To ensure the accuracy of the diaries, at the end of the study participants were asked about their experiences and potential inaccuracies in their records. They were explicitly told they would not be penalized for potential inaccuracies, as recommended by Reis and Wheeler (1991).

#### Friendship Quality

Friendship quality was measured as described in Study 1. The questionnaire showed good internal reliability (α = .83).

## Results

### Preliminary Analyses

The correlations were calculated in the same manner as in Study 1. Table 4 presents the means and standard deviations, as well as the correlations between the study variables. The within-person correlations are shown above the diagonal; the between-person correlations are shown below the diagonal; intraclass correlations are shown on the diagonal.

**Table 4.** Means, Standard Deviations, and Correlations of Study 2 Variables.

Variables	1	2	3	4	5
1. Avoidance with best friend	.21*	.39***	−.07	.07	−.22**
2. Anxiety with best friend	.18*	.29**	−.18**	−.28***	−.53***
3. Self-disclosure	.03	−.08	.18	.53****	.19**
4. Emotional support-seeking	.00	−.16*	.30***	.33***	.34***
5. Friendship quality	−.10	−.26***	.17*	.19**	.31***
*M*	2.00	2.25	2.79	2.92	3.99
*SD*	1.03	.82	.76	.83	.34

*Note*. This table shows the within-person (above diagonal), between-person (below diagonal), and intraclass (on diagonal) correlations.

* *p* < .05. **p < .01. ***p < .001.

### Model Specification

**Figure 2.**
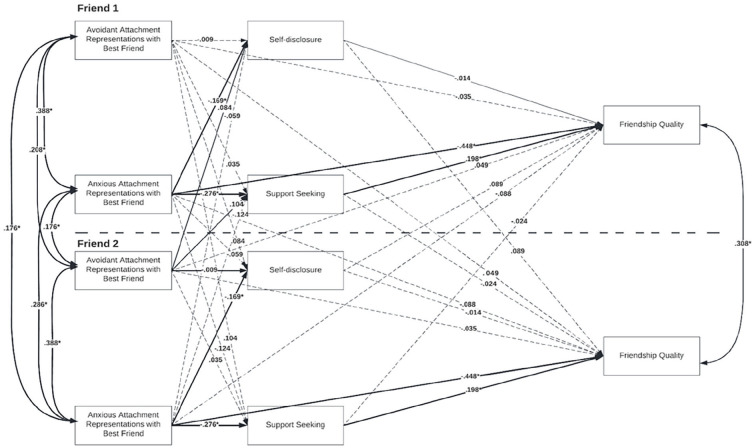
APIMeM of Study 2. *Note*. APIMeM of best friends’ attachment representations and friendship quality, mediated by intimacy behaviors. (Standardized) estimates are shown for all direct actor and partner effects, covariances between the predictors, and covariances between the outcome variables. The covariances between the mediators are not depicted in the model. *p < .05.

An indistinguishable APIMeM was run to examine the effects of attachment representations with best friend on friendship quality in the final questionnaire, mediated by self-disclosure and support-seeking (see Figure 2). Equality constraints were imposed on the actor effects, partner effects, predictor means and variances, mediator and outcome intercepts and residual variances, and covariances between friends’ error terms. The model was an interchangeable saturated model; therefore, no fit indices were reported.

Numerical inspection of the distributions showed no severe violations of normality (skew < 2; kurtosis < 7), but visual inspection showed that attachment representations with friend were slightly positively skewed. Therefore, we used the Yuan–Bentler correction for biased standard errors.

### Final Model Results

Table 5 presents all (un)standardized direct actor and partner effects and their corresponding 95% bootstrapped confidence intervals. Table 6 presents all (un)standardized indirect (mediated) actor and partner effects and their corresponding 95% bootstrapped confidence intervals.

**Table 5:** Direct Effects and Bootstrapped 95% Confidence Intervals From the APIMeM in Study 2.

Direct effect	Unst.	[95% CI]	Std.
*Actor avoidance*			
Actor avoidance with best friend → actor self-disclosure	−.007	[−.110, .097]	−.009
Actor avoidance with best friend → actor support-seeking	.028	[−.072, .129]	.035
Actor avoidance with best friend → actor friendship quality direct effect	−.011	[−.050, .027]	−.035
*Actor anxiety*			
Actor anxiety with best friend → actor self-disclosure	**−.156**	**[−.309, −.002]**	**−.169**
Actor anxiety with best friend → actor support-seeking	**−.277**	**[−.432, −.123]**	**−.276**
Actor anxiety with best friend → actor friendship quality direct effect	**−.184**	**[−.243, −.124]**	**−.448**
*Actor intimate behaviors*			
Actor self-disclosure → actor friendship quality	−.006	[−.068, .056]	−.014
Actor support-seeking → actor friendship quality	**.081**	**[.028, .133]**	**.198**
*Partner avoidance*			
Actor avoidance with best friend → partner self-disclosure	.062	[−.033, .156]	.084
Actor avoidance with best friend → partner support-seeking	.084	[−.018, .185]	.104
Actor avoidance with best friend → partner friendship quality direct effect	.016	[−.028, .060]	.049
*Partner anxiety*			
Actor anxiety with best friend → partner self-disclosure	−.054	[−.198, .089]	−.059
Actor anxiety with best friend → partner support-seeking	−.124	[−.272, .024]	−.124
Actor anxiety with best friend → partner friendship quality direct effect	−.036	[−.092, .020]	−.088
*Partner intimate behaviors*			
Actor self-disclosure → partner friendship quality	.040	[−.026, .106]	.089
Actor support-seeking → partner friendship quality	−.010	[−.068, .048]	−.024

*Note.* Unstandardized (Unst.) and standardized (Std.) estimates and bootstrapped confidence intervals are presented for all direct effects. Estimates in bold are statistically significant (*p* < .05).

**Table .6:** Indirect (Mediation) Effects and Bootstrapped 95% Confidence Intervals From the APIMeM in Study 2.

Effect	Unst.	[95% CI]	Std.
*Actor avoidance*			
Actor avoidance with best friend → actor friendship quality total effect	−.007	[−.045, .031]	−.023
Actor avoidance with best friend → actor friendship quality total indirect effect	.004	[−.007, .015]	.012
Actor avoidance with best friend → actor self-disclosure → actor friendship quality	.000	[−.001, .001]	.000
Actor avoidance with best friend → partner self-disclosure → actor friendship quality	.002	[−.003, .008]	.007
Actor avoidance with best friend → actor support-seeking → actor friendship quality	.002	[−.006, .011]	.007
Actor avoidance with best friend → partner support-seeking → actor friendship quality	−.001	[−.006, .004]	−.003
*Actor anxiety*			
Actor anxiety with best friend → actor friendship quality total effect	**−.206**	**[−.261, −.151]**	**−.503**
Actor anxiety with best friend → actor friendship quality total indirect effect	**−.022**	**[−.043, −.001]**	**−.055**
Actor anxiety with best friend → actor self-disclosure → actor friendship quality	.001	[−.009, .011]	.002
Actor anxiety with best friend → partner self-disclosure → actor friendship quality	−.002	[−.009, .005]	−.005
Actor anxiety with best friend → actor support-seeking → actor friendship quality	**−.022**	**[−.043, −.002]**	**−.055**
Actor anxiety with best friend → partner support-seeking → actor friendship quality	.001	[−.006, .008]	.003
*Partner avoidance*			
Actor avoidance with best friend → partner friendship quality total effect	.022	[−.022, .066]	.067
Actor avoidance with best friend → partner friendship quality total indirect effect	.006	[−.005, .017]	.018
Actor avoidance with best friend → actor self-disclosure → partner friendship quality	.000	[−.004, .004]	−.001
Actor avoidance with best friend → partner self-disclosure → partner friendship quality	.000	[−.004, .004]	−.001
Actor avoidance with best friend → actor support-seeking → actor friendship quality	.000	[−.002, .002]	−.001
Actor avoidance with best friend → partner support-seeking → partner friendship quality	.007	[−.003, .016]	.021
*Partner anxiety*			
Actor anxiety with best friend → partner friendship quality total effect	−.049	[−.103, .005]	−.120
Actor anxiety with best friend → partner friendship quality total indirect effect	−.013	[−.035, .008]	−.032
Actor anxiety with best friend → actor self-disclosure → partner friendship quality	−.006	[−.017, .005]	−.015
Actor anxiety with best friend → partner self-disclosure → partner friendship quality	.000	[−.003, .004]	.001
Actor anxiety with best friend → actor support-seeking → partner friendship quality	.003	[−.013, .019]	.007
Actor anxiety with best friend → partner support-seeking → partner friendship quality	−.010	[−.025, .005]	−.024

*Note.* Unstandardized (Unst.) and standardized (Std.) estimates and bootstrapped confidence intervals are presented for all indirect effects. Estimates in bold are statistically significant (*p* < .05).

#### Actor Direct Effects

Emerging adults with more anxious attachment representations disclosed less, sought less support, and reported lower friendship quality. Participants who reported more support-seeking experienced higher friendship quality. Contrary to our expectations, avoidance with best friend did not predict self-disclosure, emotional support-seeking, or friendship quality, nor did self-disclosure predict friendship quality.

#### Partner Direct Effects

No significant partner effects emerged. Thus, anxious or avoidant attachment representations with best friend did not predict the friend’s intimacy behaviors or perceived friendship quality.

#### Mediation Effects

Significance of the mediation effects was calculated as in Study 1. Contrary to our expectations, no significant actor–partner mediation effects emerged. However, a small significant actor–actor mediation effect was found. The association between actor anxiety and actor friendship quality was mediated by actor’s support-seeking. Emerging adults who had more anxious attachment representations with their best friend sought less support from their friend, which resulted in lower friendship quality.

## Summary

The goal of the second study was to examine whether attachment with a best friend predicted friendship quality, and whether this effect was mediated by intimacy behaviors (self-disclosure and support-seeking). Consistent with our hypotheses and Study 1, we found that higher attachment anxiety predicted lower friendship quality, which was mediated by actor’s support-seeking. Furthermore, we found that anxious participants disclosed less and sought less support from their friend. Contrary to our predictions and the results of Study 1, we found no effects of attachment avoidance. We also found no partner effects.

## General Discussion

The associations between attachment representations, intimacy behaviors, and friendship quality were examined in two studies with independent samples. Study 1 showed that anxious and avoidant attachment representations with parents predicted friendship quality in emerging adulthood, through their effects on attachment representations with a best friend. In Study 2, this finding was partially replicated, as anxious attachment representations with a best friend predicted friendship quality. Moreover, we found that anxious participants showed less self-disclosure and support-seeking and that the link between anxiety and friendship quality was mediated by support-seeking. Contrary to the results of Study 1, we did not find an effect of avoidant attachment representations on friendship quality in Study 2.

Consistent with previous literature, we found that attachment representations with parents and friends were related (e.g., Bohn et al., 2023; Furman et al., 2002; Pierce & Lydon, 2001). The small to moderate correlations between attachment anxiety and avoidance with parents and friend support the idea of a hierarchical structure, with overlap and also differences between attachments to parents and friends (Sibley & Overall, 2008). Interestingly, we found a stronger continuity effect for anxiety than for avoidance, which is similar to findings by Dugan et al. (2022). This suggests that attachment anxiety might be more stable across relationships than avoidance. Avoidance reflects a working model about others and might thus be more relationship-specific, as an individual might believe that their parents are available but not their friends, for example. Anxiety, on the other hand, reflects a working model about the self and might thus be more stable across relationships, as it reflects whether individuals consider themselves as worthy of love and support at all.

We also found that attachment with parents predicted friendship quality through attachment with best friend, in line with previous findings of direct effects between parental attachment and friendship quality (Furman, 2001; Zimmermann, 2004) or friend attachment and friendship quality (Özen et al., 2010). When controlling for attachment representations with best friend, we found no significant relation between attachment representations with parents and friendship quality. However, we did find a significant association between attachment representations with parents and representations with best friend, and attachment representations with best friend and friendship quality. Our findings support the continuity perspective, as individuals with less avoidant and anxious parental attachment representations also reported less avoidant and anxious attachment representations with their best friend, and experienced higher friendship quality.

We partially replicated this finding in Study 2, as we found that individuals higher in attachment anxiety experienced lower friendship quality. Moreover, they disclosed less and sought less emotional support. This is consistent with the theoretical predictions of attachment theory (Bartholomew & Horowitz, 1991) as well as with previous empirical findings (Florian et al., 1995; Markiewicz et al., 2001; Özen et al., 2010).

Interestingly, we did not replicate the association between attachment avoidance on friendship quality in Study 2. We expected avoidant individuals to report lower friendship quality, as research has shown that they show lower intimacy levels (e.g., Simpson et al., 1992; Zimmermann, 2004). However, we did not find lower intimacy levels for avoidant attachment in Study 2. Although this contradicts much research on attachment avoidance (Florian et al., 1995; Özen et al., 2010; Simpson et al., 1992), it is consistent with Kerns and Stevens (1996), who found no effect of attachment style on intimacy behaviors using the RIR. One potential explanation is that Simpson et al. (1992) measured intimacy after inducing stress, whereas we and Kerns and Stevens (1996) measured intimacy in daily interactions. It has been suggested that attachment-related differences in behaviors mostly occur in stressful situations (Bowlby, 1973, 1979). Avoidant individuals may thus only avoid intimacy in stressful situations, which might explain why we found no associations between attachment avoidance, intimacy, and friendship quality in Study 2. However, the question remains why an association between avoidance and friendship quality emerged in Study 1 and not in Study 2. Further research is necessary to examine when and how attachment avoidance relates to friendship quality.

Finally, our results showed no partner effects in Study 1 or Study 2. Thus, emerging adults whose friend had more anxious or avoidant attachment representations or showed less intimacy, did not experience lower friendship quality. This might have two statistical explanations. First, within-person correlations have the advantage of shared source variance and are therefore usually larger than between-person correlations. As a result, the partner effects may have been overshadowed by the relatively large actor effects. Second, we did not have adequate power for some, but not all, partner effects in Studies 1 and 2. Moreover, since friendships are voluntarily, individuals might only select and maintain friendships in which they perceive high friendship quality. They may let go of friends they perceive to be too anxious or avoidant or their intimacy too high or too low. Alternatively, there may indeed be no partner effects and how emerging adults perceive the quality of a friendship may not primarily depend on each other’s attachment representations or intimacy behaviors but rather on other things.

Although we have showed that attachment representations with parents and best friends and friendship quality are interrelated in emerging adulthood, the question remains whether friendship attachment and quality are a result of concurrent attachment with parents or that the interrelatedness is the result of the same underlying early working model. According to a prototype perspective, early working models are modified and revised based on new experiences, but they do not overwrite early working models. Rather, concurrent relationships are affected by both concurrent attachment representations and early working models formed in childhood. According to a revisionist perspective, however, these revised attachment representations do overwrite early working models and relationships are thus only affected by these concurrent attachments and not by early working models. Both models have been empirically supported and have their limitations (Fraley, Vicary and Brumbaugh, 2011; Jones et al., 2018; Pinquart et al., 2013). Interestingly, some studies have showed that earned secure individuals report higher relationship quality and more effective relationship behaviors than individuals who did not shift from insecure attachment in childhood to secure attachment in adulthood (Paley et al., 1999; Roisman et al., 2002), thereby showing that concurrent secure attachment representations may have a greater effect on relationship outcomes than early working models. It would be interesting to further examine the interrelatedness of concurrent attachment representations and friendship quality using longitudinal data on attachment security and friendship outcomes.

It would be interesting for future research to examine whether our results would generalize to other, potentially less close friends. It has been found that attachment representations to different friends are correlated, but differences persist due to, for example, personal characteristics of each friend and different expectations of “best friends” and “friends” (Bohn et al., 2023; Fraley & Dugan, 2021). It would also be interesting to examine the dyadic associations between concurrent attachment representations in romantic relationships and friendships, as research shows positive correlations between attachment representations to friends and a romantic partner (Bohn et al., 2023; Furman et al., 2002). However, dyadic studies on this topic are scarce.

In Study 2, we decided to aggregate the day-to-day assessments of self-disclosure and emotional support-seeking into two composite variables. Our intention was to create a reliable measure of the average levels of self-disclosure and emotional support-seeking in the friendship, which would not suffer from recall bias or chance fluctuations, which might have been the case if we had used a retrospective measure or measured the level of self-disclosure and emotional support on a random day. However, by aggregating the day-to-day assessments we were not able to assess within-person associations over time, which is the main advantage of a diary design. Since we did not collect our data with the intention of assessing these associations, we have relatively few datapoints compared with other experience sampling method (ESM) studies nor did we collect day-to-day data that could be used to answer our research question. For example, if we had collected ESM data on perceptions of friendship quality on more occasions or in a larger sample, we could have examined whether emerging adults perceive their friendship of higher quality on days they sought more support or disclosed more and whether this effect is moderated by attachment representations. Another interesting question would, for example, be whether emerging adults experience higher levels of positive affect and lower levels of negative affect on days where they or their best friend sought more support or disclosed more and whether this effect is moderated by attachment representations. These questions are therefore an interesting direction for future research.

Several other limitations have to be considered when interpreting the results. First, our assumptions about the direction of the effects were based on theory and previous research. This is a good approach to provide a model for future research of directional effects between attachment, intimacy behaviors, and friendship quality using experimental or longitudinal designs. However, we acknowledge that the design of the current studies and our use of concurrent data for Study 1 did not allow us to reach firm conclusions about the directionality of effects. Second, both studies relied on self-report measures. Besides the problem of shared-source variance, the attachment self-report measures have the disadvantage of differentiating people at the insecure end of the spectrum more precisely than at the secure end (Fraley et al., 2000). As our sample was relatively secure, secure individuals were not assessed with the same fidelity as insecure individuals. There is therefore a need for research to develop self-report measures that can discriminate secure people equally well, and to develop methods that eliminate shared source variance, for example, using independent raters of intimacy behaviors and friendship quality. Third, our participants chose the friend with whom they wanted to participate, which resulted in a sample consisting of best friend dyads that experienced relatively high friendship quality and secure attachment representations. Friendships are by definition voluntary relationships, and so that, emerging adults may let go of low-quality or insecure friendships, but the restricted range of the variables may have obscured some effects. Finally, our sample is predominantly female and highly educated, which potentially limits the generalizability of our findings.

Besides these limitations, our research had several strengths. First, the RIR makes it possible to measure naturally occurring intimacy behaviors. This was especially important in our study, as emerging adults’ attachment representations or perceptions of friendship quality may affect the recall of intimacy behaviors on a dispositional measure. Moreover, our study was the first to examine offline and online intimacy behaviors using the RIR. Although certain questions on the RIR may be less suited to online interactions (e.g., interaction duration), it is a strength to examine both online and offline behaviors as emerging adults increasingly use the online context to show intimacy and maintain friendships (Subrahmanyam et al., 2008). Second, we used continuous measures of relation-specific attachment representations, rather than categorical measures of global attachment. By doing so, we are the first, to our knowledge, to examine the associations between attachment representations with a best friend, parents, and friendship quality in emerging adulthood. Finally, we highlighted the importance of examining dyadic influences in friendships and thereby contributed to a better understanding of the dyadic nature of friendships.

In conclusion, our findings highlight the continuing importance of attachment representations with parents and best friend in emerging adulthood. We showed that anxious attachment representations with parents are continued with best friends and ultimately result in lower friendship quality due to decreased support-seeking. We also showed that avoidant attachment representations with parents are continued with best friends, but we found contradicting results regarding the link between avoidant attachment representations and friendship quality. Future research using multiple methods and research paradigms is necessary to understand when and how attachment relates to friendship behaviors and ultimately friendship quality. By doing so, we will enrich our understanding of the developmental significance of friendships in emerging adulthood.

## Supplemental Material

sj-docx-1-psp-10.1177_01461672231195339 – Supplemental material for Friendships in Emerging Adulthood: The Role of Parental and Friendship Attachment Representations and Intimacy Supplemental material, sj-docx-1-psp-10.1177_01461672231195339 for Friendships in Emerging Adulthood: The Role of Parental and Friendship Attachment Representations and Intimacy by Marie G. Oldeman, Antonius H. N. Cillessen and Yvonne H. M. van den Berg in Personality and Social Psychology Bulletin
