# Supplementary material for: Friendships in Emerging Adulthood: The Role of Parental and Friendship Attachment Representations and Intimacy
Source: Pers Soc Psychol Bull. 2023 Sep 5;51(4):514–29. doi: 10.1177/01461672231195339 (PMC11827279; doi:10.1177/01461672231195339)
Supplement: sj-docx-1-psp-10.1177_01461672231195339 – Supplemental material for Friendships in Emerging Adulthood: The Role of Parental and Friendship Attachment Representations and Intimacy [file sj-docx-1-psp-10.1177_01461672231195339.docx]

**Table 1**

| Direct effect | Unst. | [95% CI] | Std. |
| --- | --- | --- | --- |
| *Actor avoidance* | | | |
| Actor avoidance with father → actor avoidance with best friend | **.080** | **[.007, .153]** | **.141** |
| Actor avoidance with father → actor friendship quality | .003 | [-.020, .026] | ..013 |
| Actor avoidance with best friend → actor friendship quality | **-.203** | **[-.236, -.170]** | **-.511** |
| *Actor anxiety* | | | |
| Actor anxiety with father → actor anxiety with best friend | **.307** | **[.179, .434]** | **.301** |
| Actor anxiety with father → actor friendship quality direct effect | -.012 | [-.039, .014] | -.040 |
| Actor anxiety with best friend → friendship quality direct effect | **-.062** | **[-.093, -.031]** | **-.212** |
| *Partner avoidance* | | | |
| Actor avoidance with father → partner avoidance with best friend | .017 | [-.053, .088] | .031 |
| Actor avoidance with father → partner friendship quality direct effect | .006 | [-.016, .028] | .027 |
| Actor avoidance with best friend → partner friendship quality direct effect | -.025 | [-.056, .006] | -.063 |
| *Partner anxiety* | | | |
| Actor anxiety with father → partner anxiety with best friend | .074 | [-.018, .167] | .073 |
| Actor anxiety with father → partner friendship quality direct effect | -.025 | [-.054, .005] | -.082 |
| Actor anxiety with best friend → partner friendship quality direct effect | -.006 | [-.032, .021] | -.019 |

*Direct Effects and Bootstrapped 95% Confidence Intervals from the APIMeM with Attachment Representations to Father*

*Note.* Unstandardized (Unst.) and standardized (Std.) estimates and bootstrapped confidence intervals are presented for all direct effects. Estimates in bold are statistically significant (*p* < .05).

**Table 2**

| Effect | Unst. | [95% CI] | Std. |
| --- | --- | --- | --- |
| *Actor avoidance* | | | |
| Actor avoidance with father → actor friendship quality total effect | -.014 | [-.038, .011] | -.061 |
| Actor avoidance with father → actor friendship quality total indirect effect | **-.017** | **[-.031, -.022]** | **-.074** |
| Actor avoidance with father → actor avoidance with best friend → actor friendship quality | **-.016** | **[-.031, -.002]** | **-.072** |
| Actor avoidance with father → partner avoidance with best friend → actor friendship quality | .000 | [-.002, .001] | -.002 |
| *Actor anxiety* | | | |
| Actor anxiety with father → actor friendship quality total effect | **-.032** | **[-.056, -.007]** | **-.106** |
| Actor anxiety with father → actor friendship quality total indirect effect | **-.020** | **[-.032, -.007]** | **-.065** |
| Actor anxiety with father → actor anxiety with best friend → actor friendship quality | **-.019** | **[-.031, -.007]** | **-.064** |
| Actor anxiety with father → partner anxiety with best friend → actor friendship quality | .000 | [-.002, .002] | -.001 |
| *Partner avoidance* | | | |
| Actor avoidance with father → partner friendship quality total effect | .000 | [-.024, .025] | .002 |
| Actor avoidance with father → partner friendship quality total indirect effect | -.006 | [-.019, .008] | -.025 |
| Actor avoidance with father → actor avoidance with best friend → partner friendship quality | -.002 | [-.005, .001] | -.009 |
| Actor avoidance with father → partner avoidance with best friend → partner friendship quality | **-**.004 | [-.018, .011] | -.016 |
| *Partner anxiety* | | | |
| Actor anxiety with father → partner friendship quality total effect | **-.031** | **[-.061, -.001]** | **-.103** |
| Actor anxiety with father → partner friendship quality total indirect effect | -.006 | [-.017, .004] | -.021 |
| Actor anxiety with father → actor anxiety with best friend → partner friendship quality | -.002 | [-.010, .006] | -.006 |
| Actor anxiety with father → partner anxiety with best friend → partner friendship quality | -.005 | [-.011, .002] | -.015 |

*Indirect (Mediation) Effects and Bootstrapped 95% Confidence Intervals from the APIMeM with Attachment Representations to Father*

*Note.* Unstandardized (Unst.) and standardized (Std.) estimates and bootstrapped confidence intervals are presented for all indirect effects. Estimates in bold are statistically significant (*p* < .05).

**Table 3**

| Direct effect | Unst. | [95% CI] | Std. |
| --- | --- | --- | --- |
| *Actor avoidance* | | | |
| Actor avoidance with mother → actor avoidance with best friend | **.119** | **[.044, .194]** | **.203** |
| Actor avoidance with mother → actor friendship quality | .018 | [-.007, .042] | .078 |
| Actor avoidance with best friend → actor friendship quality | **-.201** | **[-.234, -.168]** | **-.511** |
| *Actor anxiety* | | | |
| Actor anxiety with mother → actor anxiety with best friend | **.489** | **[.316, .662]** | **.427** |
| Actor anxiety with mother → actor friendship quality direct effect | -.027 | [-.066, .013] | -.079 |
| Actor anxiety with best friend → friendship quality direct effect | **-.062** | **[-.096, -.028]** | **-.210** |
| *Partner avoidance* | | | |
| Actor avoidance with mother → partner avoidance with best friend | -.047 | [-.104, .009] | -.081 |
| Actor avoidance with mother → partner friendship quality direct effect | .013 | [-.010, .036] | .058 |
| Actor avoidance with best friend → partner friendship quality direct effect | -.026 | [-.058, .006] | -.066 |
| *Partner anxiety* | | | |
| Actor anxiety with mother → partner anxiety with best friend | .016 | [-.094, .125] | .014 |
| Actor anxiety with mother → partner friendship quality direct effect | -.001 | [-.033, .030] | -.004 |
| Actor anxiety with best friend → partner friendship quality direct effect | -.015 | [-.045, .015] | -.050 |

*Direct Effects and Bootstrapped 95% Confidence Intervals from the APIMeM with Attachment Representations to Mother*

*Note.* Unstandardized (Unst.) and standardized (Std.) estimates and bootstrapped confidence intervals are presented for all direct effects. Estimates in bold are statistically significant (*p* < .05).

**Table 4**

| Effect | Unst. | [95% CI] | Std. |
| --- | --- | --- | --- |
| *Actor avoidance* | | | |
| Actor avoidance with mother → actor friendship quality total effect | -.005 | [-.032, .022] | -.021 |
| Actor avoidance with mother → actor friendship quality total indirect effect | **-.023** | **[-.038, -.008]** | **-.098** |
| Actor avoidance with mother → actor avoidance with best friend → actor friendship quality | **-.024** | **[-.039, -.009]** | **-.104** |
| Actor avoidance with mother → partner avoidance with best friend → actor friendship quality | .001 | [-.001, .003] | .005 |
| *Actor anxiety* | | | |
| Actor anxiety with mother → actor friendship quality total effect | **-.057** | **[-.094, -.021]** | **-.169** |
| Actor anxiety with mother → actor friendship quality total indirect effect | **-.031** | **[-.051, -.010]** | **-.090** |
| Actor anxiety with mother → actor anxiety with best friend → actor friendship quality | **-.030** | **[-.051, -.010]** | **-.090** |
| Actor anxiety with mother → partner anxiety with best friend → actor friendship quality | .000 | [-.002, .001] | -.001 |
| *Partner avoidance* | | | |
| Actor avoidance with mother → partner friendship quality total effect | .020 | [-.006, .045] | .086 |
| Actor avoidance with mother → partner friendship quality total indirect effect | .006 | [-.006, .019] | .028 |
| Actor avoidance with mother → actor avoidance with best friend → partner friendship quality | -.003 | [-.007, .001] | -.013 |
| Actor avoidance with mother → partner avoidance with best friend → partner friendship quality | .010 | [-.002, .021] | .041 |
| *Partner anxiety* | | | |
| Actor anxiety with mother → partner friendship quality total effect | -.009 | [-.039, -.020] | -.028 |
| Actor anxiety with mother → partner friendship quality total indirect effect | -.008 | [-.025, .009] | -.024 |
| Actor anxiety with mother → actor anxiety with best friend → partner friendship quality | -.007 | [-.022, .008] | -.021 |
| Actor anxiety with mother → partner anxiety with best friend → partner friendship quality | -.001 | [-.008, .006] | -.003 |

*Indirect (Mediation) Effects and Bootstrapped 95% Confidence Intervals from the APIMeM with Attachment Representations to Mother*

*Note.* Unstandardized (Unst.) and standardized (Std.) estimates and bootstrapped confidence intervals are presented for all indirect effects. Estimates in bold are statistically significant (*p* < .05).
